# Long-term caries prevention of dental sealants and fluoride varnish in children with autism spectrum disorders: a retrospective cohort study

**DOI:** 10.1038/s41598-022-12176-7

**Published:** 2022-05-19

**Authors:** Araxi Balian, Guglielmo Campus, Giuliana Bontà, Marcella Esteves-Oliveira, Claudia Salerno, Silvia Cirio, Valeria D’Avola, Maria Grazia Cagetti

**Affiliations:** 1 Department of Biomedical, Surgical and Dental Sciences, University of Milan, Via Beldiletto 1, 20142 Milan, Italy; 2 Department of Restorative, Preventive and Pediatric Dentistry, University of Bern, Freiburgstrasse 7, 3012 Bern, Switzerland; 3 Department of Pediatric, Preventive Dentistry and Orthodontics, School of Dentistry, Sechenov University, 119991 Moscow, Russia; 4 Department of Surgery, Microsurgery and Medicine Sciences, School of Dentistry, University of Sassari, Viale San Pietro 3/c, 07100 Sassari, Italy

**Keywords:** Paediatric research, Epidemiology

## Abstract

The aim was to compare two strategies for caries prevention in children with Autism Spectrum Disorders (ASDs). Participants were retrospectively retrieved and divided in two groups. Group one had first permanent molars treated with fluoride varnishes, FA group (*n* = 92, 9.43 ± 2.44 years) whilst the second, with dental sealant plus fluoride varnishes, FA + S group (*n* = 140, 7.77 ± 2.57 years). Logistic and multivariate analysis were run to evaluate the caries incidence, the retention rate of sealants, and background factors associated with caries risk over a period of at least 11 years. Survival rates from dental caries were statistically significantly higher in the FA + S group compared to the FA group (LogRank test *p* < 0.01). Dental sealant plus fluoride varnish played as a protective factor towards the development of caries (HR = 0.25 _95%_CI = 0.00/0.55 and HR = 0.34 _95%_CI = 0.00/0.66 in the upper right and left first molars; HR = 0.32 _95%_CI = 0.00/0.66 and HR = 0.26 _95%_CI = 0.00/0.58 in the lower right and left first molars). Dental sealants retention rate was high, ranging between 58.02% and 64.29%. No baseline variable was statistically significantly associated to the risk of caries development. Combined dental sealant and fluoride varnish application was more effective in reducing caries risk in first permanent molars of ASDs children than fluoride varnish alone. This preventive strategy should be therefore routinely applied in high caries risk patients as ASDs children.

## Introduction

Many developed countries, albeit showing a decrease in caries prevalence in time, also demonstrate an unequal skewed distribution of caries figures and severity; a large proportion of caries lesions still remains untreated^1^. Worst oral health conditions are described in children from low-income families^2^. The occlusal surface of first permanent molars is the most prone to caries in children with and without disability^3–5^. Their increased exposure to the disease is linked to several factors: their posterior position in the mouth, increasing the difficulty of cleaning, the presence of pits and fissures, favoring the accumulation of plaque, and the fact that they are often equivocally considered primary teeth by parents/guardians^4,6^.

Autism Spectrum Disorders (ASDs) are lifelong neurodevelopmental disorders involving persistent challenges in social interaction, speech and nonverbal communication, and restricted and repetitive behaviors^7^. Children with ASDs have shown a poorer oral health status, hence representing a population at high risk for caries and gingivitis^8–10^. ASDs children are greatly challenged when facing new experiences such as the dental environment, which is of particular concern due to the presence of several noises, smells, and visual stimuli that might exacerbate fear and anxiety^11^. These concerns may lead parents to avoid regular dental examinations, preventive procedures and treatments^12^.

In the occlusal surface of first permanent molars, two strategies are recognized as effective in preventing caries lesions: pit and fissure sealant and fluoride varnish^13,14^. Dental sealants provide a mechanical protection against caries by sealing pits and fissures, turning highly retentive occlusal surfaces into much smoother ones, making them easier to clean and avoiding accumulation of plaque^13^. Slightly elevated levels of fluoride in the oral environment are essential in order to prevent enamel dissolution, reduce caries development, and promote remineralization of hard dental tissues making them more resistant to acid attack. Different fluoride-releasing products and materials are available to obtain this goal^16,17^. Fluoride varnishes provide a chemical protection, preventing caries through high fluoride concentration and a prolonged contact time with dental surfaces^17^. The mechanisms of action involve both inhibition of dental demineralization and enhancing of remineralization through increase of fluoride uptake by dental hard tissues as well as formation of calcium fluoride reservoirs, which in turn makes enamel and dentin more resistant to acids attacks^18^. The effectiveness of sealants and fluoride varnishes for controlling caries compared to no intervention has been clearly demonstrated, while the relative effectiveness of combining the two interventions remains unclear^13,19^.

The aim of the present paper was to evaluate whether the application of pit and fissure sealants combined with fluoride varnish is more effective than the application of solely fluoride varnish in preventing caries lesions development in the occlusal surface of first permanent molars in children with ASDs. The null hypothesis was that the caries increment in the first permanent molars would not differ significantly applying the two procedures. Demographic and clinical characteristics of patients at first dental examination were also investigated as potential protective and/or risk factors for caries development.

## Materials and methods

### Study design, sample selection and procedure

A retrospective, parallel cohort study was designed and registered at Clinical Trial.gov (NCT05146479). The study was approved by the Ethics committee Board of San Paolo Hospital, Milan, Italy (approval number 546/2021). This is a human observational study and as such was conformed to STROBE (STrengthening the Reporting of OBservational studies in Epidemiology) guidelines (Supplementary Table S1)^20^. The ethical principles stated in the Declaration of Helsinki were followed.

Considering an expected proportion of 0.57 first permanent molars free from caries due to fluoride varnish alone, a sample size of 80 teeth in each group was found in order to obtain a 90% power, at a significance level (alpha) of 0.05 (two-tailed)^21^.

The study involved ASD subjects, who have received dental care at the Dental Service for ASD children of San Paolo Hospital from January 2006 to June 2021. The primary outcome was caries survival rates of first permanent molars between subjects who received a fluoride varnish alone or combined with pit and fissure resin sealants. The secondary outcome was to evaluate if patient’s variables recorded at the first dental examination, as for example age, number of dental caries lesions and cooperation level, were positively or negatively associated with first permanent affected molars rates overtime.

The inclusion criteria were children diagnosed with ASDs: age less than 14 years at the first oral examination, follow-up equal or longer than 10 years, written declaration of informed consent signed by parents/guardians. Only children that at the first dental examination showed at least one completely erupted first permanent molar without any caries lesion assessed using the Decay, Missing, Filled Tooth index were enrolled^22^. The exclusion criteria consisted of: incomplete clinical records, missing five or more dental check-ups over the follow-up period, oral or dental care provided in sedation or general anesthesia, follow-up inferior to 10 years.

**Figure 1:**
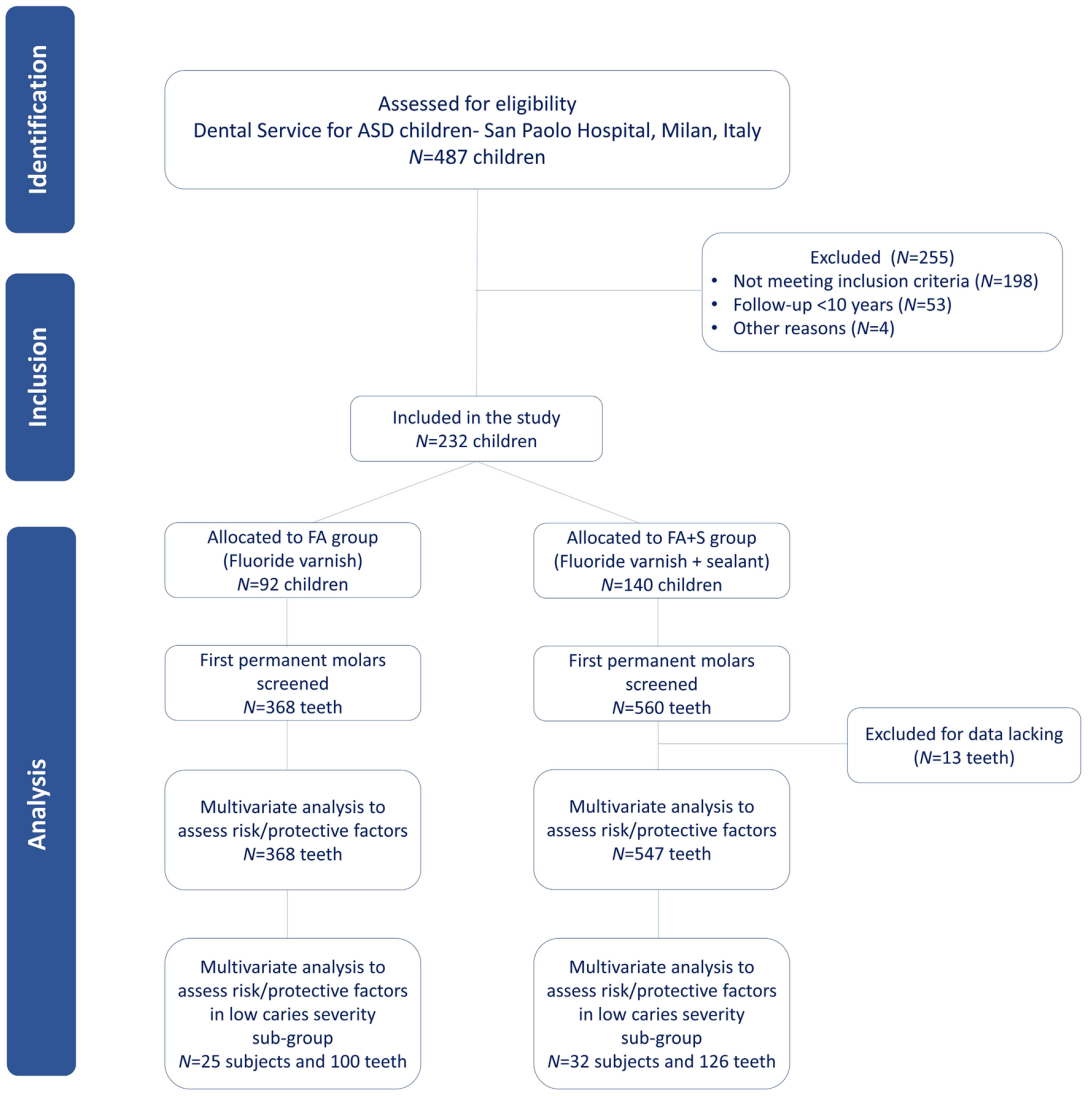
Study flow-chart according to STROBE (Strengthening the Reporting of Observational Studies in Epidemiology) statement.

Initially, 487 clinical charts were screened for eligibility of which 255 were excluded because they did not meet the inclusion criteria (*n* = 198), had a follow-up shorter than 10 years (*n* = 53), and other reasons (*n* = 4) as shown in Fig. 1. The final sample consisted 232 clinical charts of patients, divided into two parallel cohort groups according to the preventive strategy they undertook. The first group (FA group), received the application of 5% sodium fluoride (NaF) varnishes (*n* = 92), and the second group (FA + S group), received the same fluoride varnish combined with light cure resin sealants onto erupted first permanent molars (*n* = 140). Overall, 928 first permanent molars were screened and 13 were excluded from FA + S group due to incomplete data.

The preventive protocol routinely applied from 2006 at the study center in ASDs children includes: the use of visual supports before the first dental examination in order to allow the children to become familiar with the dental environment, quarterly check-ups including professional oral hygiene and application of 5% NaF varnish on primary and permanent dentitions, and finally the application of light cure dental sealants on first permanent molars.

During the introductory visit, all children/parents/caregivers received oral hygiene instructions and dietary recommendations. A toothpaste with at least 1000 ppm of fluoride was also recommended. All dental procedures, including first dental examination, follow-up check-ups, application of a fluoride varnish and dental sealants were performed by at least two second-year and/or third-year post-graduate students in Pediatric Dentistry with at least one-year training to treat patients with special needs.

### Fluoride varnish protocol (FA group)

Children whose cooperation was too poor to perform the procedure and whose parents refused the application of dental sealants and/or that denied the protective stabilization, received solely the fluoride varnish application.

The fluoride varnish application procedure was the following: excess plaque was removed using a rotary brush mounted on a low-speed handpiece with a prophylaxis paste without fluoride or, in case of poor cooperation, with cotton gauzes and a 0.12% solution of chlorhexidine gluconate; teeth were then isolated by means of cotton rolls and/or gauzes; 5% NaF varnish (Colgate Duraphat varnish, 50 mg/mL, Colgate-Palmolive Ltd, UK) was applied over all teeth at a three-months interval, including first permanent molars, following manufacture’s instruction, using a single dose of 0.40 mL, raspberry flavor. Parents/caregivers were instructed to make sure their children did not eat or drink after the application of varnish for at least an hour.

### Fluoride varnish and dental sealants protocol (FA + S group)

Patients in FA + S group, in addition to the above-mentioned procedures, received the application of pit and fissure sealants onto first permanent molars as soon as the occlusal surface was fully erupted. After tooth cleaning and isolation, pits and fissures of first permanent molars were conditioned by etching with 37% phosphoric acid (H_3_PO_4_) for 30 s, then thoroughly rinsed and dried. After this, a thin layer of a white photopolymerizable resin-based sealant without fluoride (Concise1930, 3 M ESPE, Seefeld, Germany) was placed and cured. Finally, if necessary, occlusal adjustments were performed. Fluoride varnish was then applied over all teeth, including the sealed ones, following the same protocol described for FA group.

### Assessment of dental sealants retention and dental caries presence

First permanent molars were regularly clinically checked for caries lesion detection and sealant’s retention assessment at each follow-up. Sealant retention was coded as retained when the material was fully present or partially present on the occlusal surfaces; and totally lost, when no trace of the material was observed^23^. Caries presence was evaluated according to the caries associated with restorations and sealant scores of the ICDAS-II visual classification criteria as follows: no change at surface level was considered sound, any modification at the surface related to caries was recorded as caries presence^24^. First permanent molars that present modification at surface level, and those with totally lost sealants at the follow-ups were considered as censored and excluded from the analysis.

### Data collection and statistical analysis

The following data were retrospectively and anonymously retrieved from the dental charts and input into a Microsoft Excel 2020 spreadsheet (Microsoft Corporation, Washington, USA): sex, age, number of primary and permanent teeth affected by caries, level of cooperation using the Frankl Scale and systemic diseases other than autism at the first dental visit; type of preventive treatment undergone; number of first permanent molars affected by caries during the follow-up period; number of dental sealants retained overtime^25^.

Means, standard deviations and skewness were calculated for continuous variables; in case of high skewness, data were logarithmic transformed. In the survival analysis, an event was defined as the change of status at tooth level, *i.e*. the development of a new dental caries lesion. The Kaplan–Meier estimator was applied to estimate the survival fraction of first permanent molars as the change of status (sound vs. affected by caries) and of dental sealants onto first permanent molars (present *vs* lost after the first application) during the follow-up period. Cox Proportional Hazards multivariate logistic models were run to assess factors associated with the change of status in the two cohorts examined and in the sub-groups with a low caries severity level (1–3 lesions) from both cohorts. Estimates are reported in the hazard ratio (HR) along with their respective 95% confidence interval (_95%_CI). Dummy variables were created and ran in the multivariate logistic models in case a confounding effect was played by one or more variables. Statistical analysis was performed using XLSTAT 2021.4 (Addinsoft, New York, USA) for Microsoft Excel. For all statistical analyses, the statistical significance was set at 5% (*p* < 0.05).

## Results

Overall, 914 first permanent molars were included for statistical analysis during a follow-up period ranging between 11 and 15 years (Fig. 1).

The majority of children in both groups were males, 62 in the FA group and 114 in the FA + S group, and more than 70% of all children had no systemic diseases except for ASDs (Table 1). Age and number of caries teeth at the first examination were statistically significant (t-test, p < 0.01) higher in FA group (9.43 ± 2.44 years; 3.90 ± 3.68 caries) compared to FA + S group (7.77 ± 2.57 years; 1.69 ± 3.02 caries). The cooperation level showed no statistically significant differences between the groups (χ^2^_3_ = 3.16; *p* > 0.05) as displayed in Table 1.

**Table 1:** Description of the sample enrolled at the first dental visit.

	FA group	FA + S group
	(*n* = 92)	(*n* = 140)
	*Mean* ± *SD*	
Age at baseline (years)	9.43 ± 2.44	7.77 ± 2.57
	*Unpaired* *t-test* *p* < *0.01*	
Number of caries at baseline	3.90 ± 3.68	1.69 ± 3.02
	*Unpaired* *t-test* *p* < *0.01*	
	*Frequency* *n* (*%*)	
**Caries severity**		
Caries free (no lesions)	21 (22.83)	81 (57.86)
Low severity (1–3 lesions)	25 (27.17)	33 (23.57)
High severity (≥ 4 lesions)	46 (50.00)	26 (18.57)
	*χ*^*2*^_(*2*)_ = *33.45* *p* < *0.01*	
**Systemic disease**		
No systemic disease	69 (75.00)	122 (87.14)
At least 1 systemic disease	21 (22.83)	8 (5.71)
More than 1 systemic disease	2 (1.17)	10 (7.14)
	*Fisher* *Exact* *Probability* *Test* *p* < *0.01*	
**Sex**		
Female	30 (32.61)	26 (18.57)
Male	62 (67.39)	114 (81.43)
	*χ*^*2*^_*(1*)_ = *5.23* *p* = *0.02*	
**Behaviour assessment**		
Completely uncooperative	26 (28.26)	49 (35.00)
Reluctant	29 (31.52)	34 (24.29)
Cooperative	22 (23.91)	27 (19.29)
Completely cooperative	15 (16.30)	30 (21.43)
	*χ*^*2*^_(*3*)_ = *3.16* *p* = *0.37*	

As all continuous variables recorded at the first examination had a skewed distribution, a logarithmic transformation was run to perform survival and linear regression analyses.

After the follow-up period, the overall number of first permanent molars still free from caries was significantly higher in group FA + S than in FA group (χ^2^_(1)_ range = 37.21 − 49.39; *p* < 0.01), and this trend was confirmed for each permanent molar (Supplementary Table S2): 56.52% (FA) and 93.57% (FA + S) for the upper right molar; 55.43% (FA) and 90.44% (FA + S) for the upper left molar; 54.35% (FA) and 93.53% (FA + S) for the lower left molar and finally 52.17% (FA) and 91.60% (FA + S) for the lower right molar. The overall rate of dental sealants survived after the follow-up period that never underwent a second application ranged between 58.02% in the first right lower molar and 64.29% in the first upper right molar (Supplementary Table S2).

**Figure 2:**
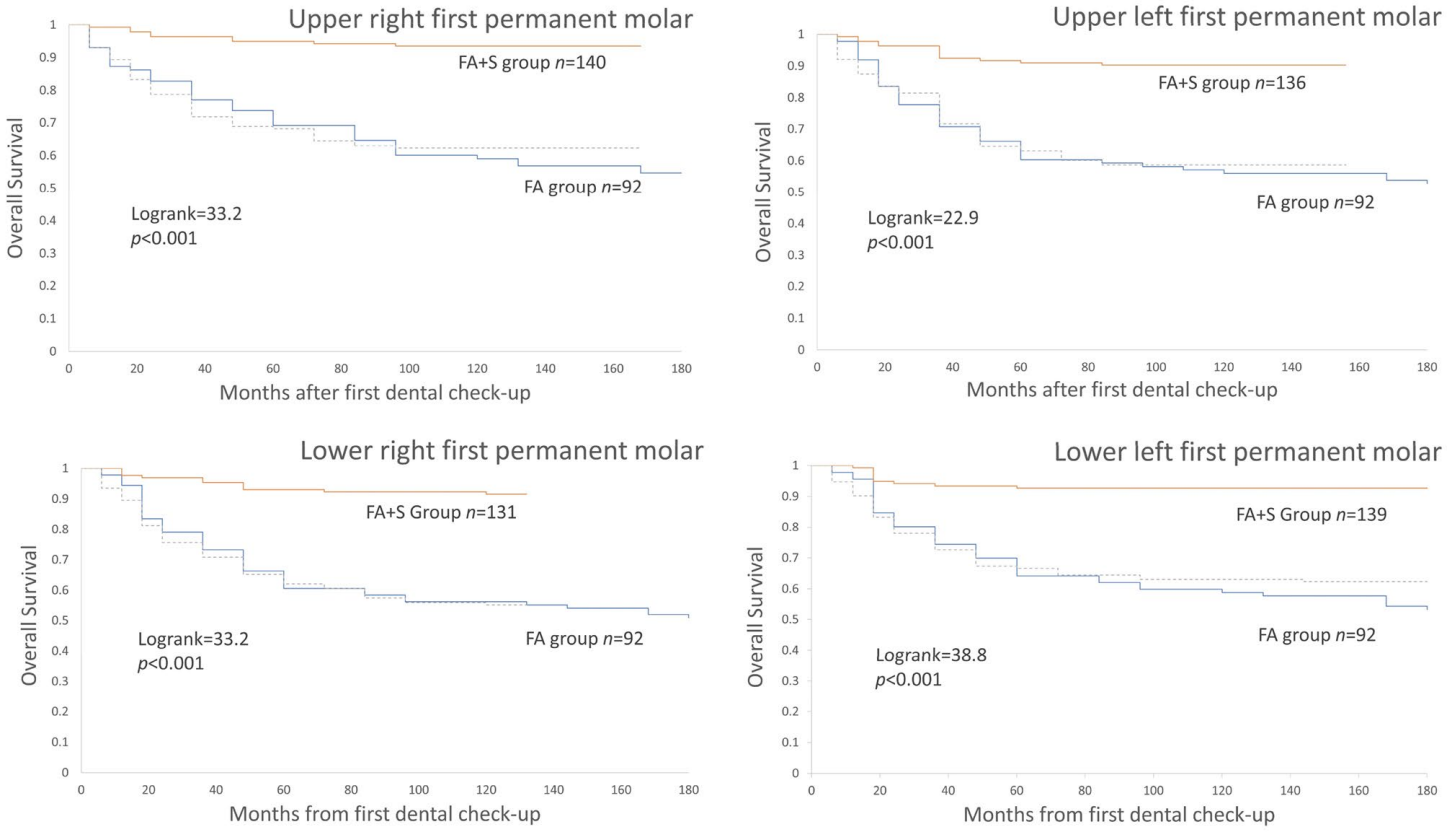
Kaplan–Meier survival curves illustrating the significance of fluoride varnish (FA group) and fluoride varnish together with fissure sealants (FA + S group) in the incidence of dental caries for each first permanent molar. Dotted lines represent Kaplan–Meier survival curves of fissure sealants for each tooth.

A statistically significant difference was found in the survival rate of each first permanent molar during the follow-up period between FA and FA + S groups (LogRank test *p* < 0.01) as shown in Fig. 2.

For all first molars, the survival curves in the two groups with the passage of time tend to move away from each other, with the curves of all first molars in the FA group showing the greatest reduction of survived teeth.

The multivariate analysis (Table 2) displays that FA + S treatment was significantly associated with a reduction in the caries risk rate for all first molars (HR = 0.25 _95%_CI = 0.00/0.55 in upper right and HR = 0.34 _95%_CI = 0.00/0.66 in upper left; HR = 0.32 _95%_CI = 0.00/0.66 in lower right and HR = 0.26 _95%_CI = 0.00/0.58 in lower left).

**Table 2:** Cox multiple logistic regression estimates of the change of status (sound *vs* affected by caries) at tooth level in FA and FA + S groups.

	Bivariate analysis		Multivariate analysis	
Covariate	Coefficient (SD)	Hazard ratio (_95%_CI)	Coefficient (SD)	Hazard ratio (_95%_CI)
**Upper right first permanent molar**				
*N* *obs* *232* *log* *Likehood* *402.09 − 437.00* *AIC* = *404.09 − 439.00* *χ*^*2*^ = *0.55 − 35.46* *p* < *0.01 − 0.46* *N* *obs* *232* *log* *Likehood* *379.78* *AIC* = *395.78* *χ*^*2*^ = *57.77* *p* < *0.01*				
Type of treatment (*F* + *S*)	− 1.93 (0.37)	0.15 (0.00 − 0.30)	− 1.38 (0.40)	0.25 (0.00 − 0.55)**
Age at first dental visit	0.21 (0.05)	1.23 (1.11 − 1.36)**	0.12 (0.06)	1.13 (1.00 − 1.28)*
Systemic diseases	− 0.17 (0.24)	0.84 (0.53 − 1.35)	− 0.41 (0.31)	0.67 (0.36 − 1.22)
Caries at baseline	0.14 (0.03)	1.15 (1.09 − 1.22)**	0.11 (0.04)	1.11 (1.03 − 1.20)**
Sex (female)	0.61 (0.30)	1.85 (1.03 − 3.31)*	0.36 (0.31)	1.43 (0.78 − 2.62)
Reluctant	0.03 (0.38)	1.03 (0.50 − 2.15)	− 0.14 (0.39)	0.87 (0.40 − 1.88)
Cooperative	0.40 (0.38)	1.48 (0.71 − 3.09)	0.26 (0.38)	1.30 (0.62 − 2.75)
Completely cooperative	− 0.79 (0.52)	0.46 (0.17 − 1.26)	− 0.69 (0.55)	0.50 (0.17 − 1.47)
**Upper left first permanent molar**				
*N* *obs* *228* *log* *Likehood* *448.96 − 497.32* *AIC* = *450.96 − 499.32* *χ*^*2*^ = *0.12 − 28.78* *p* < *0.01 − 0.73* *N* *obs* *228* *log* *Likehood* *443.15* *AIC* = *455.15* *χ*^*2*^ = *54.29* *p* < *0.01*				
Type of treatment (*F* + *S*)	− 1.59 (0.32)	0.20 (0.00 − 0.38)**	− 1.09 (0.34)	0.34 (0.00 − 0.66)**
Age at first dental visit	0.19 (0.05)	1.21 (1.09 − 1.34)**	0.15 (0.06)	1.16 (1.03 − 1.31)*
Systemic diseases	0.08 (0.23)	1.08 (0.69 − 1.69)	0.02 (0.27)	1.02 (0.60 − 1.75)
Caries at baseline	0.18 (0.03)	1.20 (1.12 − 1.28)**	0.16 (0.04)	1.18 (1.09 − 1.27)**
Sex (*female*)	0.34 (0.30)	1.40 (0.78 − 2.50)	0.29 (0.31)	1.34 (0.73 − 2.48)
Reluctant	− 0.41 (0.35)	0.66 (0.34 − 1.31)	− 0.67 (0.36)	0.51 (0.26 − 1.03)
Cooperative	− 0.14 (0.35)	0.87 (0.44 − 1.72)	− 0.51 (0.36)	0.60 (0.30 − 1.22)
Completely cooperative	− 1.01 (0.50)	0.37 (0.00 − 0.97)	− 0.82 (0.51)	0.44 (0.16 − 1.20)
**Lower left first permanent molar**				
*N* *obs* *231* *log* *Likehood* *417.01 − 455.11* *AIC* = *419.01 − 457.11* *χ*^*2*^ = *0.02 − 38.12* *p* < *0.01 − 0.87* *N* *obs* *231* *log* *Likehood* *393.38* *AIC* = *405.38* *χ*^*2*^ = *61.76* *p* < *0.01*				
Type of treatment (*F* + *S*)	− 1.98 (0.37)	0.14 (0.00 − 0.29)**	− 1.33 (0.41)	0.26 (0.00 − 0.58)**
Age at first dental visit	0.16 (0.05)	1.17 (1.05 − 1.30)**	0.10 (0.06)	1.10 (0.98 − 1.25)
Systemic diseases	0.04 (0.26)	1.04 (0.63 − 1.73)	− 0.11 (0.31)	0.90 (0.49 − 1.65)
Caries at baseline	0.23 (0.03)	1.26 (1.18 − 1.35)**	0.19 (0.04)	1.21 (1.12 − 1.31)**
Sex (*female*)	0.07 (0.32)	1.07 (0.57 − 2.01)	0.08 (0.35)	1.09 (0.55 − 2.14)
Reluctant	− 0.01 (0.37)	0.99 (0.48 − 2.04)	− 0.26 (0.38)	0.77 (0.37 − 1.61)
Cooperative	0.21 (0.36)	1.23 (0.60 − 2.51)	− 0.12 (0.37)	0.89 (0.43 − 1.85)
Completely cooperative	− 0.96 (0.56)	0.38 (0.13 − 1.16)	− 0.83 (0.57)	0.43 (0.14 − 1.33)
**Lower right first permanent molar**				
*N* *obs* *224* *log* *Likehood* *448.48 − 489.70* *AIC* = *450.48 − 491.70* *χ*^*2*^ = *0.08 − 41.30* *p* < *0.01 − 0.78* *N* *obs* *224* *log* *Likehood* *431.35* *AIC* = *443.35* *χ*^*2*^ = *58.44* *p* < *0.01*				
Type of treatment (*F* + *S*)	− 1.74 (0.34)	0.18 (0.00 − 0.34)**	− 1.16 (0.38)	0.32 (0.00 − 0.66)**
Age at first dental visit	0.11 (0.05)	1.12 (1.01 − 1.23)*	0.07 (0.06)	1.08 (0.96 − 1.21)
Systemic diseases	− 0.29 (0.30)	0.74 (0.42 − 1.33)	− 0.48 (0.34)	0.62 (0.32 − 1.21)
Caries at baseline	0.24 (0.03)	1.28 (1.19 − 1.36)**	0.19 (0.04)	1.21 (1.12 − 1.31)**
Sex (*female*)	− 0.09 (0.32)	0.91 (0.49 − 1.71)	− 0.01 (0.34)	0.99 (0.51 − 1.93)
Reluctant	− 0.14 (0.36)	0.87 (0.43 − 1.77)	− 0.35 (0.37)	0.71 (0.34 − 1.45)
Cooperative	0.17 (0.35)	1.18 (0.59 − 2.37)	− 0.08 (0.37)	0.93 (0.45 − 1.90)
Completely cooperative	− 0.48 (0.45)	0.62 (0.25 − 1.50)	− 0.39 (0.46)	0.68 (0.28 − 1.67)

*p-value < 0.05 **p-value < 0.01.

Caries prevalence recorded at the first dental examination was significantly associated to an increased risk of caries of each first permanent molar (p < 0.01). The higher the age at the first dental visit, the higher the risk of caries in upper first molars (HR = 1.13 _95%_CI = 1.00/1.28 for the right and HR = 1.16 _95%_CI = 1.03/1.31 for the left). The presence of systemic diseases other than ASD and the level of cooperation at the first dental examination were not associated with the risk of developing new caries. The process of assessment of the multivariate models allows to discover that sex and age at the first examination played a confounding effect when tested in the bivariate analysis for upper right molar and lower molars. A dummy variable with the two variables was then created and ran, but no significant association were found.

The caries figures (Table 1) were different between the two groups (FA and FA + S), so it was decided to run a Cox multiple logistic regression to estimate the change of status (sound *vs* affected by caries) at tooth level in low caries severity (1–3 lesions) sub-groups of FA and FA + S groups (Table 3). The FA + S treatment was able to reduce the risk of new lesions in the lower molars.

**Table 3:** Cox multiple logistic regression estimates of the change of status (sound *vs* affected by caries) at tooth level in low caries severity (1–3 lesions) cohort of FA and FA + S groups.

Covariate	Coefficient (SD)	Hazard ratio (_95%_CI)
**Upper right first permanent molar**		
*N* *obs* *57* *log* *Likehood* *74.57* *AIC* = 74.57 *χ*^*2*^ = *3.76* *p* = *0.44*		
Type of treatment (*F* + *S*)	− 0.40 (0.65)	0.67 (0.23 − 1.95)
No systemic disease	− 0.59 (0.67)	0.56 (0.18 − 1.68)
Cooperative	0.12 (0.66)	1.12 (0.38 − 3.31)
Sex (*female*)	0.86 (0.64)	2.36 (0.83 − 6.77)
**Upper left first permanent molar**		
*N* *obs* *57* *log* *Likehood* *77.42* *AIC* = *81.42* *χ*^*2*^ = *6.48* *p* = *0.04*		
Type of treatment (*F* + *S*)	− 0.51 (0.65)	0.60 (0.21 − 1.75)
Sex (*female*)	1.33 (0.63)	3.78 (1.34 − 10.67)
**Lower left first permanent molar**		
*N* *obs* *57* *log* *Likehood* = *82.15* *AIC* = *82.15* *χ*^*2*^ = *6.20* *p* = *0.10*		
Type of treatment (*F* + *S*)	− 1.56 (0.70)	0.21 (0.07 − 0.66)*
No systemic disease	0.73 (0.80)	2.07 (0.56 − 7.68)
Non cooperative	− 0.53 (0.63)	0.59 (0.21 − 1.67)
**Lower right first permanent molar**		
*N* *obs* *55* *log* *Likehood* *88.88* *AIC* = *88.88* *χ*^*2*^ = *6.10* *p* = *0.19*		
Type of treatment (*F* + *S*)	− 1.22 (0.65)	0.29 (0.10 − 0.85)
No systemic disease	1.59 (1.07)	4.90 (0.84 − 28.46)
Non cooperative	− 0.10 (0.64)	0.91 (0.32 − 2.61)
Sex (*female*)	0.12 (0.64)	1.13 (0.39 − 3.26)

## Discussion

This is, to author’s best knowledge, the first long-term retrospective cohort study investigating caries survival of first permanent molars achieved by the application of both dental sealant and fluoride varnish in children with autism spectrum disorders.

Pit and fissure sealants combined with fluoride varnish quarterly applied revealed to be significantly more effective in long-term caries prevention than the sole application of fluoride varnish: ASDs children in FA + S group had a significant lower caries rate than children in FA group. This finding is in accordance with a previous paper that supports the fact that an additional caries protection effect may be gained by adding dental sealant to fluoride varnish^21^. Sealants supply a physical barrier to dental plaque accumulation in pits and fissures, while fluoride varnish, due to its high concentration of fluoride, promotes enamel reminaralization by increasing fluoride uptake as well as forming calcium fluoride reservoirs^2,13,18,19,26^. These last two mechanisms may have produced an additional caries prevention effect both in teeth with fully retained sealant and in those where sealants were partially lost, increasing the success of the double strategy. This approach might be very helpful in children with special needs, such as ASD children, in whom the risk of caries is often high due to poor hygiene habits and a diet rich in fermentable carbohydrates^8,9,12^.

Children with ASDs have been already proved to have a high risk of caries due to multiple factors related to their behavioral and communicative impairment, such as poor oral hygiene, increased consumption of sugary foods, and alteration of oral microbiota^10,27^. Preventive treatment in children with ASDs is advocated to reduce the likelihood of development of dental caries and periodontal disease as well as to avoid complex dental therapies. Moreover, dental treatments in these children are challenging due to their poor cooperation even if properly prepared and supported^11,28^. Fissure sealants, together with fluoride varnish, provide a long-term beneficial/protective effect reducing the risk of caries in all first permanent molars along the follow-up period. Data suggest that this approach may be recommended routinely in children with ASDs, since it can be performed quite simply, it does not require the use of aerosol generating instruments and it is suitable in both primary and secondary dental care settings^13,14,29,30^.

The retention rate of dental sealant in FA + S group proved to be strong in the mid-term, as survival rates ranged between 58% (11-years follow-up) and 64% (14-year follow-up). Other studies reported higher retention rate, ranging between 73.7% and 91.4%, but a shorter follow-up period (2–3 years) and healthy and cooperative children were considered^21,29^.

The subgroup analysis in children with low caries severity confirmed that FA + S group gained a higher protective effect from dental caries compared to FA group, although it was significant only for the lower molars. This result is supported by the current body of evidence underlining that effectiveness of dental sealants is related to the overall caries risk and it is generally less pronounced in low caries risk children compared to high caries risk patients^13,19,29,30^. In addition, first mandibular molars were found to be statistically more affected by caries than their maxillary counterparts^31^. This finding can be explained by the low number of sites with dental plaque found in maxillary molars; moreover, the mandibular molars show an earlier eruption than their maxillary counterparts, but a longer time need to fully erupt and, as consequence, they might be exposed to a higher caries risk^32^.

Age, level of cooperation, presence of systemic diseases other than ASD and caries severity at the first dental examination together with sex were not associated with caries development in first permanent molars during the long follow-up period. Sex and age played a confounding effect in the bivariate analysis, but the dummy variable obtained as their sum revealed to be not significantly associated with caries development.

Within the limit of the study, this data suggests that preventive treatment in children with ASDs is effective and can be performed efficiently in many patients regardless their behavioral impairment and oral/general health status. It must be said that participants were all treated in a highly specialized secondary care center delivering dental treatment in children with ASDs. This might explain why the effectiveness of the preventive treatment was not influenced by baseline patients’ variables. The application of a strict and frequent recall program may also explain this successful result, despite literature does not demonstrate which is the most suitable interval for dental preventive recalls^33,34^.

The retrospective study design accounts for inner limitations. Treatments were performed in a clinical setting without a pre-established rationale for scientific purpose, increasing the influence of possible confounders. Another limitation is the absence of a no-treatment control group that would be useful to assess absolute effectiveness of the both preventive strategies applied. Nevertheless, current evidence shows that fluoride varnishes and dental sealants are both effective in caries prevention compared to no preventive treatment, although a higher cost-effectiveness is reported for fluoride varnishes, especially when used in community and primary care settings^13,29,35^. A further limitation is that the two cohorts of participants considered were not matched for some baseline variables: FA group had significantly higher age, caries severity and number of systemic diseases other than ASD than FA + S group. However, regression analysis showed that the same variables did not influence the probability of developing new caries on permanent molars overtime. It is also interesting to underline that subjects assigned to FA group were those whose parents did not give their consent to perform dental sealants and/or the advanced behavioral management technique where needed. This strengthened the recommendation for an early dental examination in children with ASDs for both preventive and educational purposes^36–38^.

The main strengths of this study are the long follow-up period considered (over 10 years) and the type of patients, children with ASDs, that often lack a good level of oral health. Children with ASD need a simple but effective preventive strategy to avoid complex dental treatments that are often carried-out under deep sedation or general anesthesia^27^. Furthermore, regular check-ups at short intervals allow for the creation of a routine that can improve the collaboration of these patients.

## Conclusion

Dental sealants and fluoride varnishes combined are more effective than the sole application of fluoride varnish in reducing caries risk in first permanent molars of children with ASDs. These findings suggest that this preventive strategy could be routinely applied in special needs patients regardless their level of cooperation, age and caries risk.

## Supplementary Information

Supplementary Information 1.

Supplementary Information 2.

## Data Availability

Data will be available upon request to the corresponding author.
