# Supplementary material for: Long-term caries prevention of dental sealants and fluoride varnish in children with autism spectrum disorders: a retrospective cohort study
Source: Sci Rep. 2022 May 19;12:8478. doi: 10.1038/s41598-022-12176-7 (PMC9119978; doi:10.1038/s41598-022-12176-7)
Supplement: Supplementary file 1 — Supplementary Information 1. [file 41598_2022_12176_MOESM1_ESM.docx]

**Supplementary File 1**

**Long-term caries prevention of dental sealants and fluoride varnish in children with Autism Spectrum Disorders: a retrospective cohort study**

Araxi Balian^a°*^, Guglielmo Campus^b,c,d°^, Giuliana Bontà^a^, Marcella Esteves-Oliveira^b^, Claudia Salerno^a^, Silvia Cirio^a^, Valeria D’Avola^a^, Maria Grazia Cagetti^a°^

**TABLE S1**. STROBE (Strengthening the Reporting of Observational Studies in Epidemiology) Statement—Checklist reporting list of items that were included in the study*.*

|  | Item No | Recommendation | Page No |
| --- | --- | --- | --- |
| **Title and abstract** | 1 | (*a*) Indicate the study’s design with a commonly used term in the title or the abstract | p.1 |
|  |  | (*b*) Provide in the abstract an informative and balanced summary of what was done and what was found |  |
| Introduction | | | |
| Background/rationale | 2 | Explain the scientific background and rationale for the investigation being reported | pp.3-4 |
| Objectives | 3 | State specific objectives, including any prespecified hypotheses | p.4 |
| Methods | | | |
| Study design | 4 | Present key elements of study design early in the paper | p.4 |
| Setting | 5 | Describe the setting, locations, and relevant dates, including periods of recruitment, exposure, follow-up, and data collection | pp.4-6 |
| Participants | 6 | (*a*) Give the eligibility criteria, and the sources and methods of selection of participants. Describe methods of follow-up | p.4 |
|  |  | (*b*) For matched studies, give matching criteria and number of exposed and unexposed |  |
| Variables | 7 | Clearly define all outcomes, exposures, predictors, potential confounders, and effect modifiers. Give diagnostic criteria, if applicable | p.6 |
| Data sources/ measurement | 8 | For each variable of interest, give sources of data and details of methods of assessment (measurement). Describe comparability of assessment methods if there is more than one group | p.6 |
| Bias | 9 | Describe any efforts to address potential sources of bias | p.7 |
| Study size | 10 | Explain how the study size was arrived at | p.7 |
| Quantitative variables | 11 | Explain how quantitative variables were handled in the analyses. If applicable, describe which groupings were chosen and why |  |
| Statistical methods | 12 | (*a*) Describe all statistical methods, including those used to control for confounding | pp.6-7 |
|  |  | (*b*) Describe any methods used to examine subgroups and interactions |  |
|  |  | (*c*) Explain how missing data were addressed |  |
|  |  | (*d*) If applicable, explain how loss to follow-up was addressed |  |
|  |  | (*e*) Describe any sensitivity analyses |  |
| Results | | |  |
| Participants | 13 | (a) Report numbers of individuals at each stage of study—eg numbers potentially eligible, examined for eligibility, confirmed eligible, included in the study, completing follow-up, and analysed | p.7 and Fig.1 |
|  |  | (b) Give reasons for non-participation at each stage |  |
|  |  | (c) Consider use of a flow diagram |  |
| Descriptive data | 14 | (a) Give characteristics of study participants (eg demographic, clinical, social) and information on exposures and potential confounders | p.7, Fig.1, and Table.1 |
|  |  | (b) Indicate number of participants with missing data for each variable of interest |  |
|  |  | (c) Summarise follow-up time (eg, average and total amount) |  |
| Outcome data | 15 | Report numbers of outcome events or summary measures over time | p.8 and Table.S2 |

| Main results | 16 | (*a*) Give unadjusted estimates and, if applicable, confounder-adjusted estimates and their precision (eg, 95% confidence interval). Make clear which confounders were adjusted for and why they were included | pp.8-9 and Table.2 |
| --- | --- | --- | --- |
|  |  | (*b*) Report category boundaries when continuous variables were categorized |  |
|  |  | (*c*) If relevant, consider translating estimates of relative risk into absolute risk for a meaningful time period |  |
| Other analyses | 17 | Report other analyses done—eg analyses of subgroups and interactions, and sensitivity analyses | p.9 and Table.3 |
| Discussion | | | |
| Key results | 18 | Summarise key results with reference to study objectives | p.9 |
| Limitations | 19 | Discuss limitations of the study, taking into account sources of potential bias or imprecision. Discuss both direction and magnitude of any potential bias | p.11 |
| Interpretation | 20 | Give a cautious overall interpretation of results considering objectives, limitations, multiplicity of analyses, results from similar studies, and other relevant evidence | pp.9-12 |
| Generalisability | 21 | Discuss the generalisability (external validity) of the study results | pp.10-11 |
| Other information | | | |
| Funding | 22 | Give the source of funding and the role of the funders for the present study and, if applicable, for the original study on which the present article is based | p.13 |
