# Supplementary material for: Long-term caries prevention of dental sealants and fluoride varnish in children with autism spectrum disorders: a retrospective cohort study
Source: Sci Rep. 2022 May 19;12:8478. doi: 10.1038/s41598-022-12176-7 (PMC9119978; doi:10.1038/s41598-022-12176-7)
Supplement: Supplementary file 2 — Supplementary Information 2. [file 41598_2022_12176_MOESM2_ESM.docx]

**Supplementary File 2**

**Long-term caries prevention of dental sealants and fluoride varnish in children with Autism Spectrum Disorders: a retrospective cohort study**

Araxi Balian^a°*^, Guglielmo Campus^b,c,d°^, Giuliana Bontà^a^, Marcella Esteves-Oliveira^b^, Claudia Salerno^a^, Silvia Cirio^a^, Valeria D’Avola^a^, Maria Grazia Cagetti^a°^

**TABLE S2.** Survival rates of first permanent molars from dental caries in FA and FA+S groups as well as survival rate of dental sealants in FA+S group over a follow-up period of 15 years.

|  |  | *Survival from dental caries* | | *Survival of sealants* |
| --- | --- | --- | --- | --- |
|  |  | **FA group** | **FA+S group** | **FA+S group** |
| **Type of molar** | **Status** | n (%) | n (%) | n (%) |
| Upper right primary molar | sound | 52 (56.52) | 131 (93.57) | 90 (64.29) |
|  | affected | 40 (43.48) | 9 (6.43) | 50 (35.71) |
|  | total | 92 (100.00) | 140 (100.00) | 140 (100.00) |
|  | χ^2^_(1)_=45.74 *p*<0.01 | | |  |
| Upper left primary molar | sound | 51 (55.43) | 123 (90.44) | 83 (61.03) |
|  | affected | 41 (44.57) | 13 (9.56) | 53 (38.97) |
|  | total | 92 (100.00) | 136 (100.00) | 136 (100.00) |
|  | χ^2^_(1)_=37.21 *p*<0.01 | | |  |
| Lower left primary molar | sound | 50 (54.35) | 130 (93.53) | 88 (63.31) |
|  | affected | 42 (46.65) | 9 (6.47) | 51 (36.69) |
|  | total | 92 (100.00) | 139 (100.00) | 139 (100.00) |
|  | χ^2^_(1)_=49.39 *p*<0.01 | | |  |
| Lower right primary molar | sound | 48 (52.17) | 120 (91.60) | 76 (58.02) |
|  | affected | 44 (47.83) | 11 (8.40) | 55 (41.98) |
|  | total | 92 (100.00) | 131 (100.00) | 131 (100.00) |
|  | χ^2^_(1)_=45.22 *p*<0.01 | | |  |
